# A patient perspective of complementary and integrative medicine (CIM) for migraine treatment: a social media survey

**DOI:** 10.1186/s12906-021-03226-0

**Published:** 2021-02-10

**Authors:** Deena E. Kuruvilla, Amit Mehta, Nidhi Ravishankar, Robert P. Cowan

**Affiliations:** 1 Yale School of Medicine, New Haven, USA; 2 Stanford University School of Medicine, Stanford, USA

## Abstract

**Abstract:**

To survey persons with migraine who use social media about Complementary and Integrative Medicine (CIM) for the treatment of migraine.

**Background:**

CIM encompasses medical treatments that are not part of but are used in concert with mainstream medicine. Between 28 and 82% of people with migraine use non-drug approaches, and approximately 50% of people with migraine do not discuss non-drug treatments with their healthcare providers (HCPs). It is important for providers to be conversant with CIM treatments and the available evidence-based data. To further this effort, people with migraine were surveyed directly through social media to identify CIM practices in which they engage.

**Methods:**

In collaboration with the American Migraine foundation (AMF) and Yakkety Yak, a digital marketing agency, we conducted a cross-sectional survey study. Participants were recruited from the Move Against Migraine (MAM) Facebook group which has 20,000+ members. The goals of the survey were to assess the attitudes toward CIM among this group, to identify which CIM modalities are being used and to determine what patients considered to be the most effective CIM modalities. While Yakkety Yak posted the survey link on the group page, the survey itself was hosted on Qualtrics, a confidential survey service.

**Results:**

372 MAM members (approximately 2%) responded to the questionnaire, of which 335 reported using CIM; between 114 and 139 (34–42%) found CIM modalities to be at least mildly effective. Of note, 164 (49%) reported using cannabis derivatives or cannabinoids, specifically with, 64/164 (39%) reporting that cannabis was not effective for them.

**Conclusions:**

This study provides an initial investigation into the demographic and practice patterns of migraine patients who use CIM. While this sampling may not reflect CIM use across all individuals with migraine, it does strongly suggest the need for better education on the role of, and evidence for, CIM among headache care providers, and the need to ask patients specifically about their use of and interest in CIM.

## Background

Migraine affects 1 out of every 7 Americans annually and is 2 to 3 times more common in females than males [1]. The financial burden of migraine in the United States is estimated to be $1533 per patient annually for episodic migraine and $4144 for those with chronic migraine. Costs are higher in vulnerable or underserved populations, such as those who have low socioeconomic status, the uninsured, and the unemployed [1].

While new migraine preventive and abortive treatments are emerging, research shows that a significant percentage of our patients are looking beyond standard medical treatments and incorporating complementary and integrative medicine (CIM) [2]. According to epidemiological studies, 30–82% of people with headache use CIM approaches [3, 4], while only 43% of people with headache discuss their CIM treatments with their healthcare provider [2].

CIM is defined by the National Center for Complementary and Integrative Health (NCCIH) as treatments that are separate from mainstream medicine but may be integrated with it [2]. Mainstream medicine in migraine treatments include conservative pharmaceutical drugs such as acute abortive drugs (triptans, ergotamines etc.), preventive medications (antidepressants, beta blockers etc.) and over the counter medications (ibuprofen, Excedrin, naproxen etc. [5]).

NCCIH was previously called the National Center for Complementary and Alternative medicine (NCCAM) but was changed to NCCIH based on the escalation in use of complementary approaches by Americans. NCCIH more accurately reflects that Americans are no longer using these approaches “alternatively” but rather in conjunction with mainstream medicine [2, 3] Examples of CIM are commonly divided into two main categories: Natural products (herbs, vitamins, minerals and probiotics) and mind-body practices (yoga, acupuncture, chiropractic, meditation and massage therapy) [2, 3, 6]. Each category is further divided into a subcategory of CIM with meditation/yoga, herbal therapies, massage/chiropractic, and acupuncture being the top CIM in each category, respectively.

In 2012, the National Health Interview Survey reported on 88,962 American adults and 17,321 children and found 33.2% of adults and 11.6% of children used CIM in the previous 12 months [4]. Additionally, Americans spent $30.2 billion on complementary health approaches during the same period.

Educated women with migraine are more likely to use CIM [7]. Wells et al., used the 2007 National Health Interview Survey (*n* = 23,393) to compare CIM use between adults with and without migraine/severe headache [2]. 49.5% of patients with migraine/severe headache used at least 1 Integrative treatment in the previous 12 months compared to 33.9% of patients without migraine/severe headache [2]. Researchers noted that adults with migraine/severe headache used CIM more often for treatment because: their provider recommended it, mainstream treatment was ineffective, or mainstream treatment was too expensive [2].

Similarly, Rhee et al. used the 2012 National Health Interview Survey to estimate the prevalence rates of CIM use in adults with migraine/severe headache (*n* = 4447) and the reason behind their use (wellness, treatment or both) [6]. 41.3% of patients with migraine/severe headache stated they used CIM in the previous 12 months [6]. 29.6% used CIM for wellness only, 11.4% for treatment of migraine/severe headache and 59% for both wellness and treatment [6]. These data also show that only 31.3% of patients reported that a provider recommended CIM [8] and that fewer than 50% of adults with migraine/severe headaches discuss CIM use with their healthcare provider [2].

To our knowledge, there are no publications using social media to investigate integrative methods used by migraine patients for treatment. Yuan et al. [9] created an online research account and associated hashtags on twitter to recruit individuals to a survey about HIV clinical outcomes. They found social media to be indispensable for recruitment and found it efficient and cost-effective. Online recruitment and social media strategies are particularly used for recruiting individuals that are hard to reach, unable to spend extra time in the office to answer research questions or those hard to engage through traditional means.

The purpose of this study was to assess the attitudes toward CIM among people with migraine in a social media group, to identify which CIM modalities are being used and to determine what patients considered to be the most effective CIM modalities.

## Methods

### Study design

This was a cross-sectional web-based survey study of people with migraine who have used CIM to treat and manage migraine. This cross-sectional survey study was conducted from January 2019 to March 2019 in collaboration with the American Migraine Foundation (AMF) and Yakkety Yak. The AMF and Yakkety Yak, a digital marketing agency, launched the nationwide #MoveAgainstMigraine campaign in 2017 to mobilize and empower people living with migraine. This initiative led to the creation of the Move Against Migraine (MAM) Facebook group for migraine patients to advocate on behalf of themselves, to understand treatment options, to access resources to manage migraine symptoms, and to connect with leading doctors and researchers. During January of 2019, the group had approximately 18,000 members.

Using literature review and clinical knowledge, two board-certified headache specialists,(DEK and RPC) developed a survey instrument that included questions based on the existing literature surrounding evidence based CIM for the treatment of migraine. The first part of study questions served to obtain demographic data, confirm a diagnosis of migraine and characterize episodic versus chronic migraine. The second part of study questions focused specifically on the CIM approaches used and their perceived effectiveness. The third part of the survey focused on the credentials of the provider giving the participant CIM guidance, if they reported having guidance, and if CIM approaches are used in conjunction with mainstream medicine.

#### Survey administration

This cross-sectional study was conducted using structured surveys; the main goal being to seek input from respondents about their experiences with CIM for migraine treatment. Consent was first obtained from patients in order to participate in the survey. Anyone part of the (MAM) group was able to see the following post, and click on it if they wanted to participate:

“Are you a migraine patient over the age of 18 who is living with migraine and using integrative remedies not prescribed by your physician (acupuncture, Coenzyme Q, cannabinoids, massage therapy, etc.)? Dr. Deena Kuruvilla, a headache specialist from Yale School of Medicine, is conducting a survey on the use of integrative medicines/therapies for migraines, and we need your help. Please click here to participate.”

Yakkety Yak posted our 17-question survey to the MAM Facebook community, for 2 months with reposting of the survey every 2 weeks. The survey was voluntary, and no incentive was offered for completing it.

Permission was obtained from The AMF, Yakkety Yak and the Yale University institutional review board to post our survey on social media.

This study was submitted for review to the IRB and was granted an exemption. When the participant clicked to participate in the survey, the first screen was a consent. They clicked a box in order to obtain their permission to complete the survey. While we specifically introduced our survey with migraine and integrative medicine in our social media link, we did have participants click on the link to confirm that they have not used integrative medicine. While Yakkety Yak posted the survey link on the group page, the survey itself was hosted on Qualtrics. Yakkety Yak did not have access to the collected data.

Qualtrics is an online survey platform developed in Provo, Utah, United States to create, test and share surveys in real time. Qualtrics provides tools that can be used to configure survey properties and to customize privacy settings, so respondents cannot be tracked to an IP or email address, name, ticket number, etc., which allows for anonymous responses. With these mechanisms as well as University firewall protected equipment, patient information was protected from unauthorized access. The data collected were used to better understand the integrative treatment methods used by migraine patients and to identify which methods are felt to be the most helpful. If any data were missing from participants, the participant survey was excluded using Qualtrics. Qualtrics has software in place to prevent repeat survey submission by the same individual.

### Statistical analysis

The percentage of respondents selecting each category is reported. For a 95% confidence level, we estimated a margin of error using +/−(1/√N), where N is the sample size [10]. With our sample size of 335, we estimated a 5% margin of error. This analysis was descriptive in nature and therefore no formal hypothesis testing was conducted.

## Results

A total of 412 migraine patients responded to the survey. Of the 412 patients, only 377 (91.5%) completed the survey in its entirety. 5 (1.3%) patients were never diagnosed with migraine by a medical professional, leaving 372 (99%) clinically diagnosed with migraine. 335 out of the 372 (90%) answered yes to “Do you use Complementary and Integrative Medicine (CIM) approaches to treat migraine.” 37 (9.9%) responded no to the above question. Of the patients who use CIM, 316 (94.3%) were female and 17 (5%) were male. Further demographics can be reviewed in Table 1. 247 users (73.7%) met the International Classification of headache disorders, 3rd edition (ICHD-3) diagnosis of chronic migraine while 87 users (25.9%) met criteria for episodic migraine. Specific questions were asked in the survey to differentiate episodic migraine and chronic migraine. 291 (86.6%) patients used CIM in combination with mainstream migraine treatments. Regarding how patients are seeking guidance for their CIM treatments, 68 (20.2%) are being managed by a healthcare provider,24 (7.1%) patients utilize the internet, 12 (3.5%) seek help from a fellow person with migraine, 4 (1.1%) from a Naturopathic provider, and 193 (57.6%) from 2 or more of the above.

**Table 1:** The CARE Mnemonic may be used when used when exploring a patient’s interest in CIM

• Conventional therapy experiences	
• Avoid judgement	
• Review integrative approaches and their limitations	
• Explore why person is interested	

Patients were asked which category of CIM (Meditation, relaxation, deep breathing exercises, acupuncture, guided imagery, yoga, cognitive behavioral therapy, biofeedback, mindfulness training, craniosacral therapy, or migraine specific supplements/vitamins) they use. 19 (5.6%) patients use meditation, relaxation, breathing exercises, and/or guided imagery. 9 (2.6%) selected cognitive behavioral therapy, 2 (.59%) patients use craniosacral therapy, 4 (1.19%) use mindfulness training, 2 (.59%) selected biofeedback, 9 patients (2.6%) selected yoga, 63 (18.8%) selected vitamins, 189 (56.4%) patients answered that they use 2 or more of the above, and 45 (13.4%) selected other.

24 (5.9%) patients reported CIM treatments were very effective (50–100% reduction in headache days), 92 (22.6% selected moderately effective (10–50% reduction), 139 (34.1%) slightly effective (1–10% reduction in headache days), 76 (18.6%) selected not effective at all (no change in headache days) and 76 (18.6%) did not answer.

With respect to nutraceutical use, 4 patients (1.19%) used Riboflavin, 78 (23.2%) use Magnesium, 4 (1.19%) selected Coenzyme q10, 1 (.298%) selected Butterbur, 15 (4.47%) selected other, 209 (62%) used a combination of 2 or more. 22 patients (6.56%) did not use any vitamin. Of those who used vitamins, 12 (3.58%) patients felt the vitamins were very effective (50–100% reduction in headache days), 58 (17.3%) selected moderately effective (10–50% reduction), 132 (39.4%) slightly effective (1–10% reduction in headache days), 109 (32.5%) not effective at all (no change in headache days) and 24 (7.1%) did not answer.

Although 10.7% of patients do not use any manipulation or body-based practices for migraine prevention, most (55.5%) use 2 or more therapies in combination. Of the individual therapies, participants reported using massage therapy was most frequently used (12.5%), then chiropractic maneuvers (9.2%), next acupuncture (9.0%--note: round all numbers to the same decimal point). Craniosacral therapy was minimally used (1.2%) as well as “other” therapies (1.7%). 27 (8.0%) patients answered that manipulation and/or body-based practices were very effective (50–100% reduction in headache days), 75 (22.3%) selected moderately effective (10–50% reduction), 114 (34%) patients selected slightly effective (1–10% reduction in headache days), 81 (24.1%) patients selected this CIM was not effective at all (no change in headache days) and 38 (11.3%) did not answer.

Finally, the survey ended with two questions regarding cannabinoids and their perceived effectiveness in preventing migraine. 164 (48.9%) use cannabidiol oil (CBD) or other cannabis derivative to prevent migraine and 171 (51%) selected no to this question. 4 (2.4%) of the 164 patients found CBD or other cannabis derivatives extremely effective; 14 (8.5%) found these products very effective (50–100% reduction in headache days), 36 (21.9%) selected moderately effective (10–50% reduction); 43 (26.2%) found them slightly effective (1–10% reduction in headache days); 64 (39%) patients found CBD or other cannabis derivatives not effective at all (no change in headache days).

## Discussion

### Study findings

Our study is the first of its kind to identify social media as a vehicle to investigate common CIM approaches used by migraine patients for headache relief. There have however been other online survey studies. Lee et al. administered a 30-min self-report survey on an online migraine headache resource (Migraine in America, www.migraine.com) to investigate if CIM produced a negative life impact of headaches for chronic migraine patients [11]. They found that approximately half of the participants reported using three CIM treatments and yet, felt dissatisfied or indifferent to their treatment strategy [11]. They also found that migraine patients who use CIM were more likely to have more frequent migraine headaches [11].

With a 95% confidence interval, our study shows that a true mean lies between the 95% of the values we have acquired, and only a 5% possibility that it does not. Only 20.2% of participants in our study seek guidance for their CIM strategies specifically by a healthcare provider (MD, DO, NP, PA), while over 50% of patients seek guidance using multiple other strategies such as research the internet, guidance from a fellow migraine sufferer, or guidance from a Naturopathic provider. It is crucial that physicians query patients about their use of herbs, supplements, and vitamins with their standard treatment, provide realistic expectations, and identify potential treatment adverse effects, drug-drug interactions and existing evidence base. Patients using butterbur for example [12], must be counseled to obtain pyrrolizidine-alkaloid-free formulations due to the potential for hepatotoxicity. Liver function must be closely monitored while using this supplement.

In combination with supplements such as magnesium and riboflavin for the prevention of migraine, many patients used meditation, relaxation, acupuncture, deep breathing exercises, guided imagery, yoga, cognitive behavioral therapy, biofeedback, mindfulness training, and craniosacral therapy concurrently with the majority (56.4%) using 2 or more of these treatments.

Of those, 76.7% of respondents found the combination of CIM treatments moderately to very effective. This finding supports the existing literature that these strategies are favorably used in combination among the general population [13] and in other select populations [14–16].

The evidence for using mind-body relaxation techniques recommended by the US Headache Consortium Guidelines [17] is based on the Agency for Health Care Policy and Research Technical Review, which found relaxation training (progressive muscle relaxation, autogenic training, meditation or passive relaxation), electromyography (EMG) biofeedback, and thermal biofeedback combined with relaxation training to have high quality (Grade A) evidence from well-performed research studies for the prevention of migraine [18]. Mindfulness training always differs from meditation through the practice of both informal and formal self-reflection during the day, while meditation is defined to be during a specific time of day at a specific place.

From our study, 30.3% of patients found perceived manipulation or body-based practices such as acupuncture, chiropractic maneuvers, etc. alone provided moderately to very effective treatment.

Finally, only 30.4% of respondent patients using cannabis as a treatment for moderate/severe migraine found it moderately to very effective. As early as the third and fourth centuries BCE, Ayurvedic preparations used cannabis for “diseases of the head” like migraine [19]. It is postulated that cannabis shows potential to interrupt glutamate signaling leading to cortical spreading depression, serotonin release from platelets and cranial blood vessel dilation caused by nitrous oxide and calcitonin gene-related peptide [20].

Medical cannabis is becoming a popular addition to mainstream pharmaceutical therapies due to rapidly changing marijuana laws and increased availability throughout the country. While some studies have shown a possible benefit of cannabis for the treatment of migraine [21] and medication overuse headache [21], properly constructed placebo-controlled trials are required to determine its true efficacy and adverse effects.

If patients and providers can have shared goals about integrative medicine use in migraine, they can have an open, non-judgmental dialogue about the risks and benefits of various approaches. The C.A.R.E mnemonic [22] can be used when discussing CIM approaches with patients. Patients may have a specific perspective regarding treatment options based on their previous treatment experiences. It can be helpful to ask about their history with **c**onventional treatments while also **a**voiding judgement. When **r**eviewing integrative treatment options, it is imperative to counsel patients on their limitations so that they have appropriate expectations. Finally, in order to adequately educate patients on CIM, it is helpful to **e**xplore where the patient’s interest in CIM stems from. See Table 2.

**Table 2:**
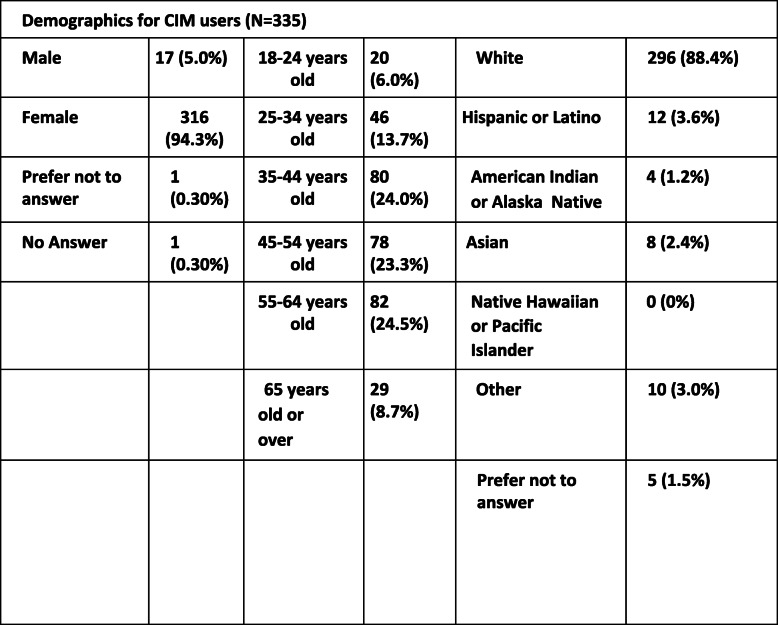
Demographic table of people with migraine who report using CIM

Many patients use CIM because it fits in with their attitudes about health [23]; addressing these beliefs, as well as potential benefits and complications, will improve patient-provider communication and more patient-centered care.

### Study limitations

The survey focused primarily on effectiveness of CIM based on frequency of headaches and not severity. Hence, this study cannot be generalized to include improvement in severity over time.

Selection bias is inherent in all social media – based studies. We were only able to capture data from those patients who are currently active in the Facebook group being polled. This limits the data applicability to the wider migraine audience, of which a small portion may be overrepresented in this group. Other biases may be introduced by unforeseen variables.

Although we had posted on the Facebook group, our findings cannot represent a true population average because not every single MAM member who uses CIM filled out the survey. Sources of bias in representing the general population include a poor survey response rate and differences in the patient characteristics of MAM compared to the general population.

Subjective biases are also inherent in self-reported outcome measures. Respondents may have misinterpreted the question and may threaten the validity and reliability of measurement. While the lines of communication were always open between respondents and the headache team to address any questions, few respondents took advantage. However, some of the advantages of self-reported outcome measures is its relatively easy collection and at a low-cost for researchers.

We cannot regulate which users take part in the survey. The survey has questions designed to obtain information from patients who have a history or diagnosis of migraine; however, this cannot be confirmed. This is an unavoidable side effect of anonymous polling and may impact the reliability of the data. Although participants were advised to complete the survey once, the number of times the survey could have been completed by an individual was not regulated since the survey was anonymous through Qualtrics. The data presented in the text as well as the tables indicate “2 or more therapies in combination”. We did not investigate which therapies were used in combinations.

This survey had not been previously validated or applied previously in studies. The study is also limited by a small sample size, a disproportionately large percentage of Caucasian female participants, and a predominantly chronic migraine population which is not representative of the larger migraine population within the United States. Many chronic migraine patients turn to CIM because they have been refractory to mainstream treatments. For this reason, the data in this study regarding patient responsiveness to CIM treatments must be interpreted with caution as many of the survey respondents may have been refractory to mainstream treatments. The responses seen in this survey may not be generalizable to the average patient.

### Future research

Larger survey studies are needed to gain a broader perspective of CIM use among people with migraine.

## Conclusion

This study uses social media to survey people with migraine and gain their perspectives on various CIM treatments. 90% of people surveyed reported using CIM for the treatment of migraine. 74% of participants met the diagnostic criteria for chronic migraine and 26% of participants met the diagnostic criteria for episodic migraine. Most patients (86%) reported using CIM in combination with mainstream migraine treatments. While this sample of people with migraine may not reflect CIM use among the general migraine population, it encourages healthcare providers to conduct solid research studies specifically using CIM for migraine treatment, increase CIM education among healthcare providers and specifically ask patients about their interest in and use of CIM.
